# Supplementary figures and images for: ARHGAP18-ezrin functions as an autoregulatory module for RhoA in the assembly of distinct actin-based structures
Source: eLife. 2024 Jan 9;13:e83526. doi: 10.7554/eLife.83526 (PMC10830128; doi:10.7554/eLife.83526)

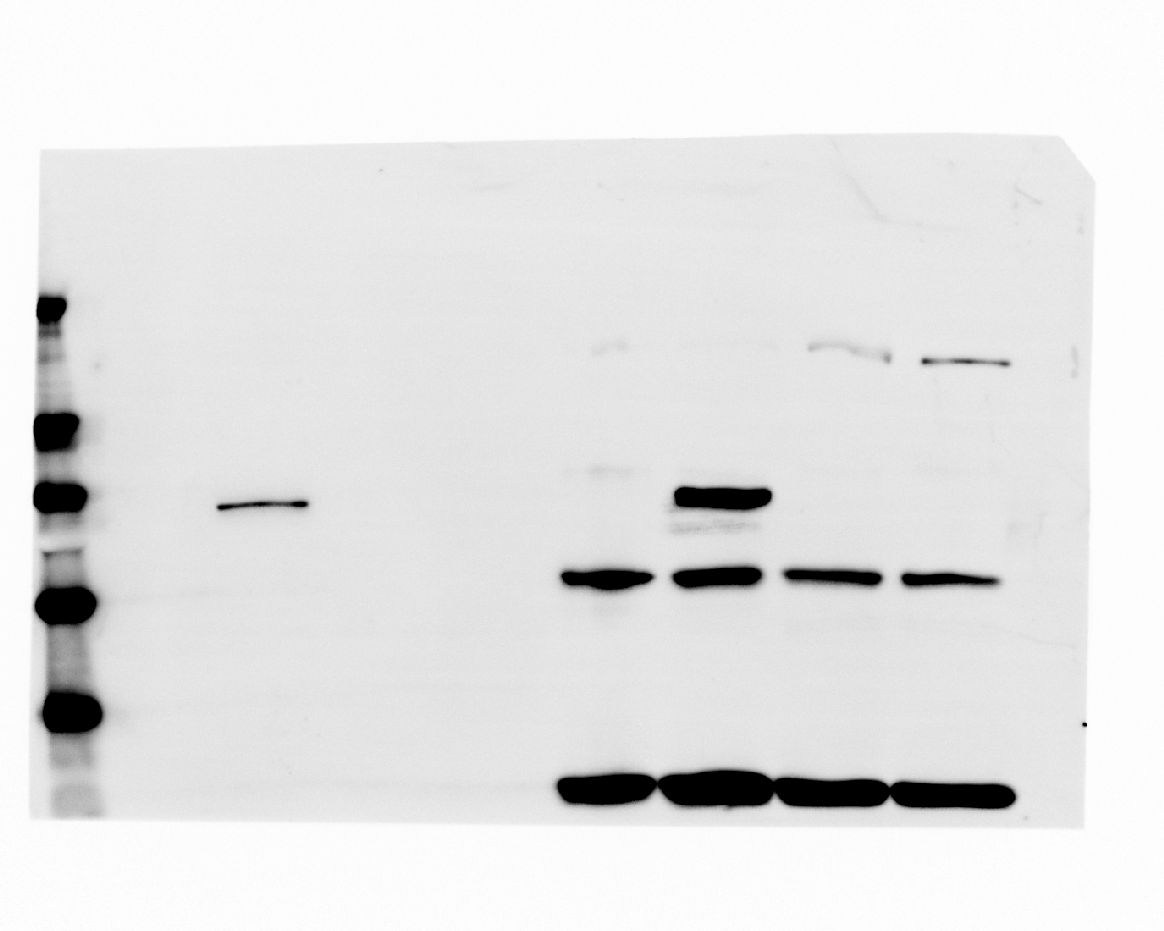

Supplement: Figure 1—source data 1. [file elife-83526-fig1-data1.zip › Figure 1-source data 1/2021-05-11 13h37m13s DyLight 680(DyLight 680).tif]

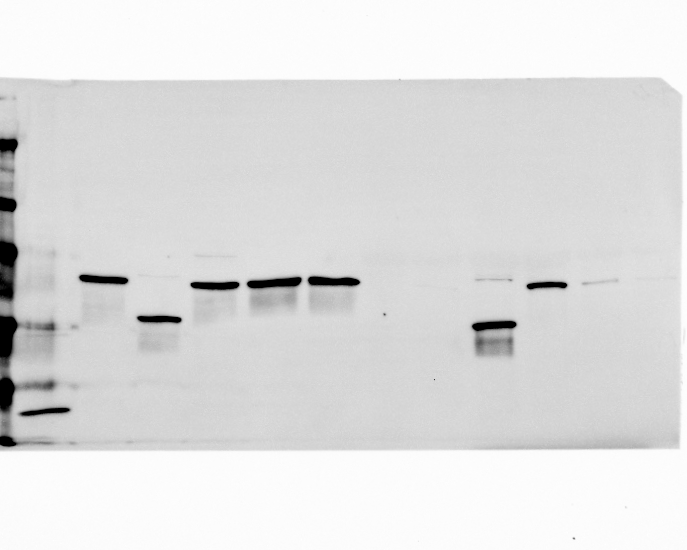

Supplement: Figure 1—source data 1. [file elife-83526-fig1-data1.zip › Figure 1-source data 1/2022-02-07 jeg3 c.sl arhgap pulldown 4 680.tif]

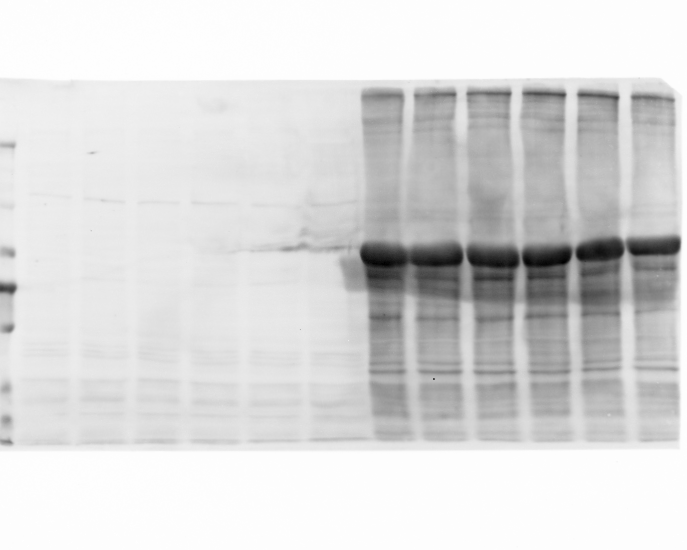

Supplement: Figure 1—source data 1. [file elife-83526-fig1-data1.zip › Figure 1-source data 1/2022-02-07 jeg3 c.sl arhgap pulldown 4 800.tif]

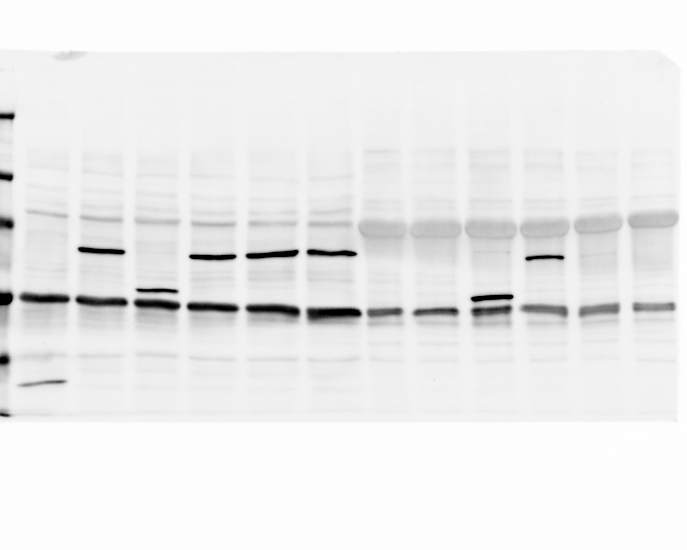

Supplement: Figure 1—source data 1. [file elife-83526-fig1-data1.zip › Figure 1-source data 1/2022-02-08 jeg3 c.sl arhgap pulldown 4 tubulin 680.tif]

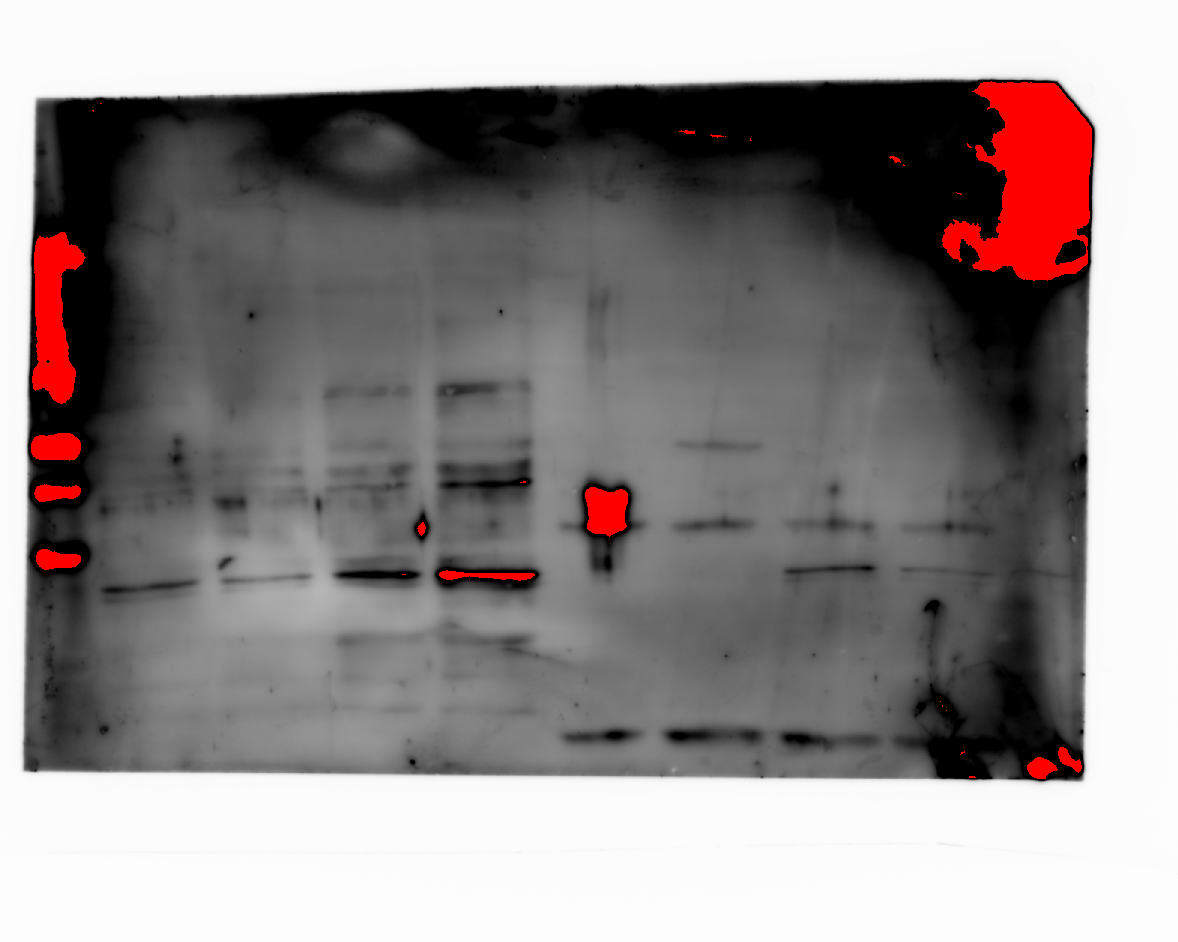

Supplement: Figure 1—source data 1. [file elife-83526-fig1-data1.zip › Figure 1-source data 1/exrin iflag arhgap18 pulldown_3(Chemiluminescence).tif]

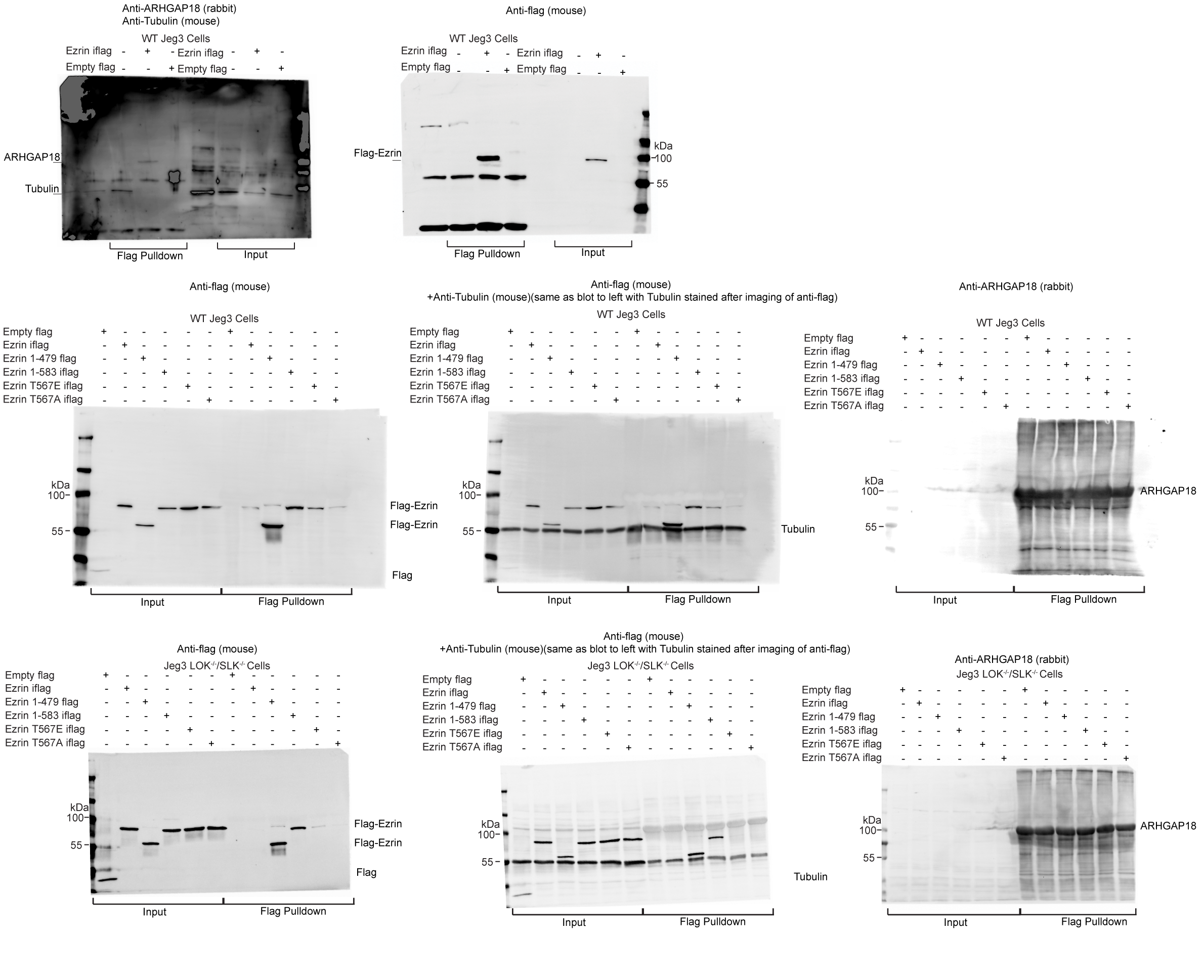

Supplement: Figure 1—source data 1. [file elife-83526-fig1-data1.zip › Figure 1-source data 1/Figure 1 VF Source Data-02.tif]

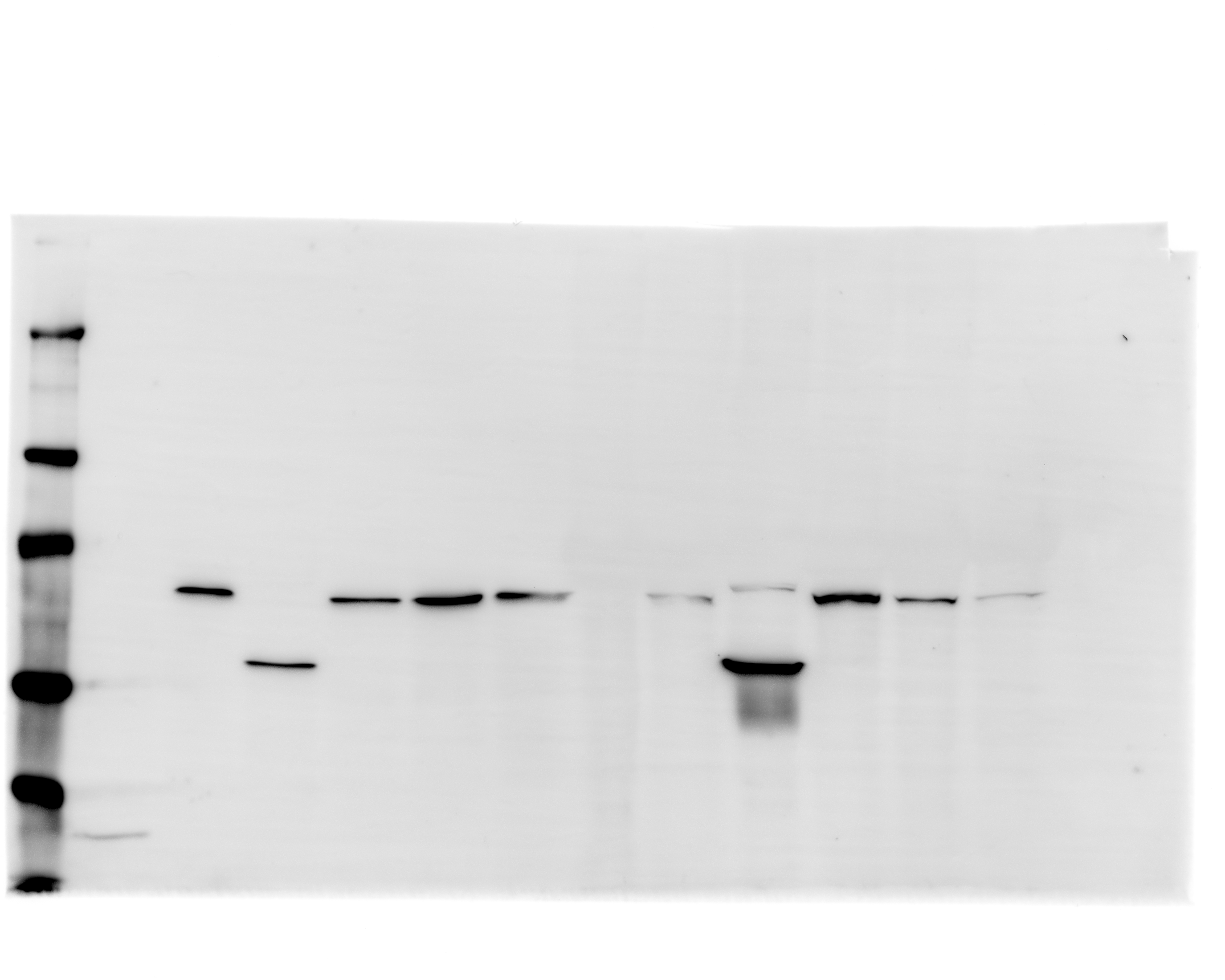

Supplement: Figure 1—source data 1. [file elife-83526-fig1-data1.zip › Figure 1-source data 1/Jeg3 WT arhgap18 pulldown-4 anti-ezrin_B.tif]

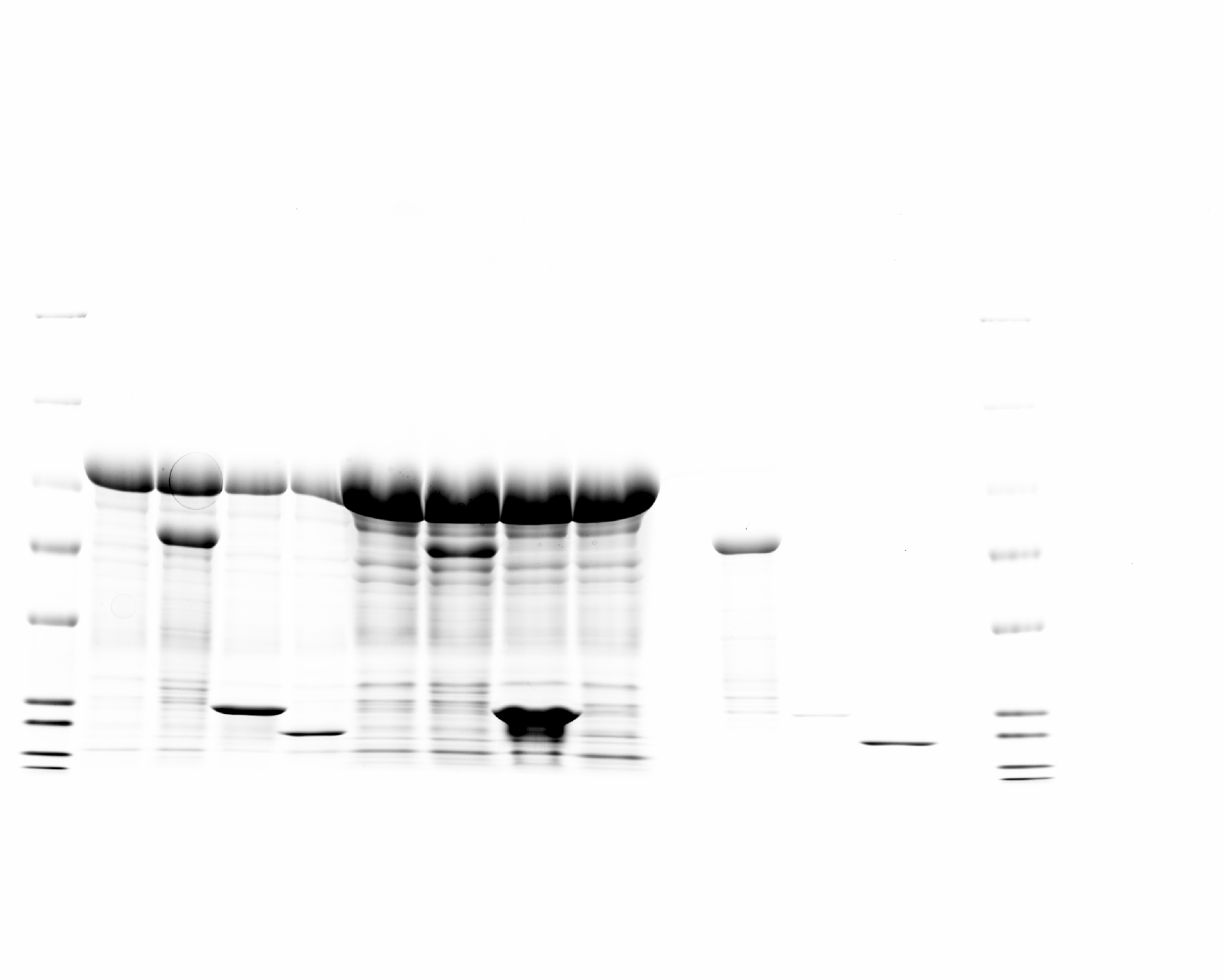

Supplement: Figure 1—figure supplement 1—source data 1. [file elife-83526-fig1-figsupp1-data1.zip › Supplemental Figure S1- source Data 1/2021-07-20 11h09m13s Coomassie Blue(Coomassie Blue).tif]

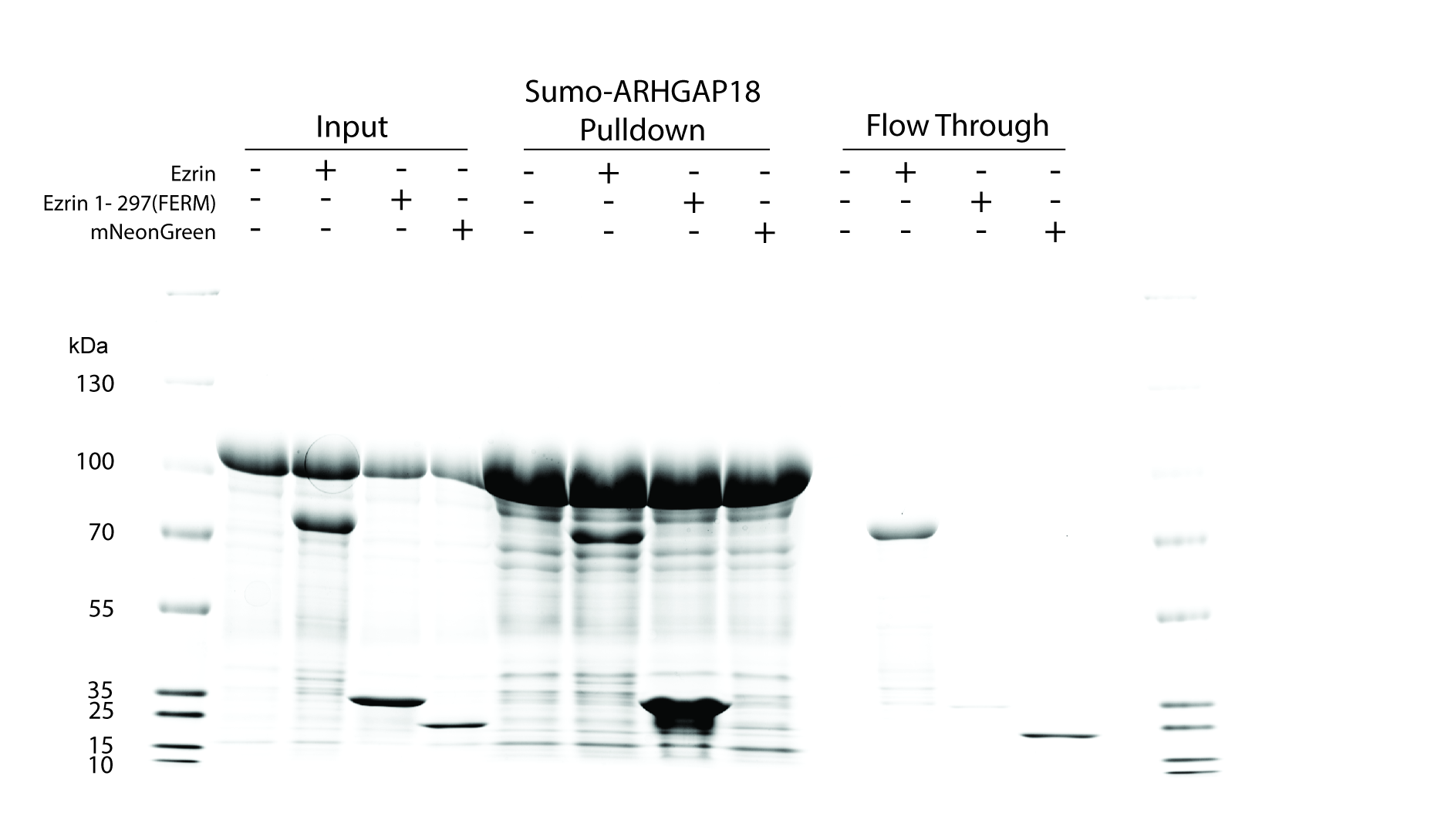

Supplement: Figure 1—figure supplement 1—source data 1. [file elife-83526-fig1-figsupp1-data1.zip › Supplemental Figure S1- source Data 1/Supplemental Figure 1 ITC FERM vs Ezrin V2-Source Data.tif]

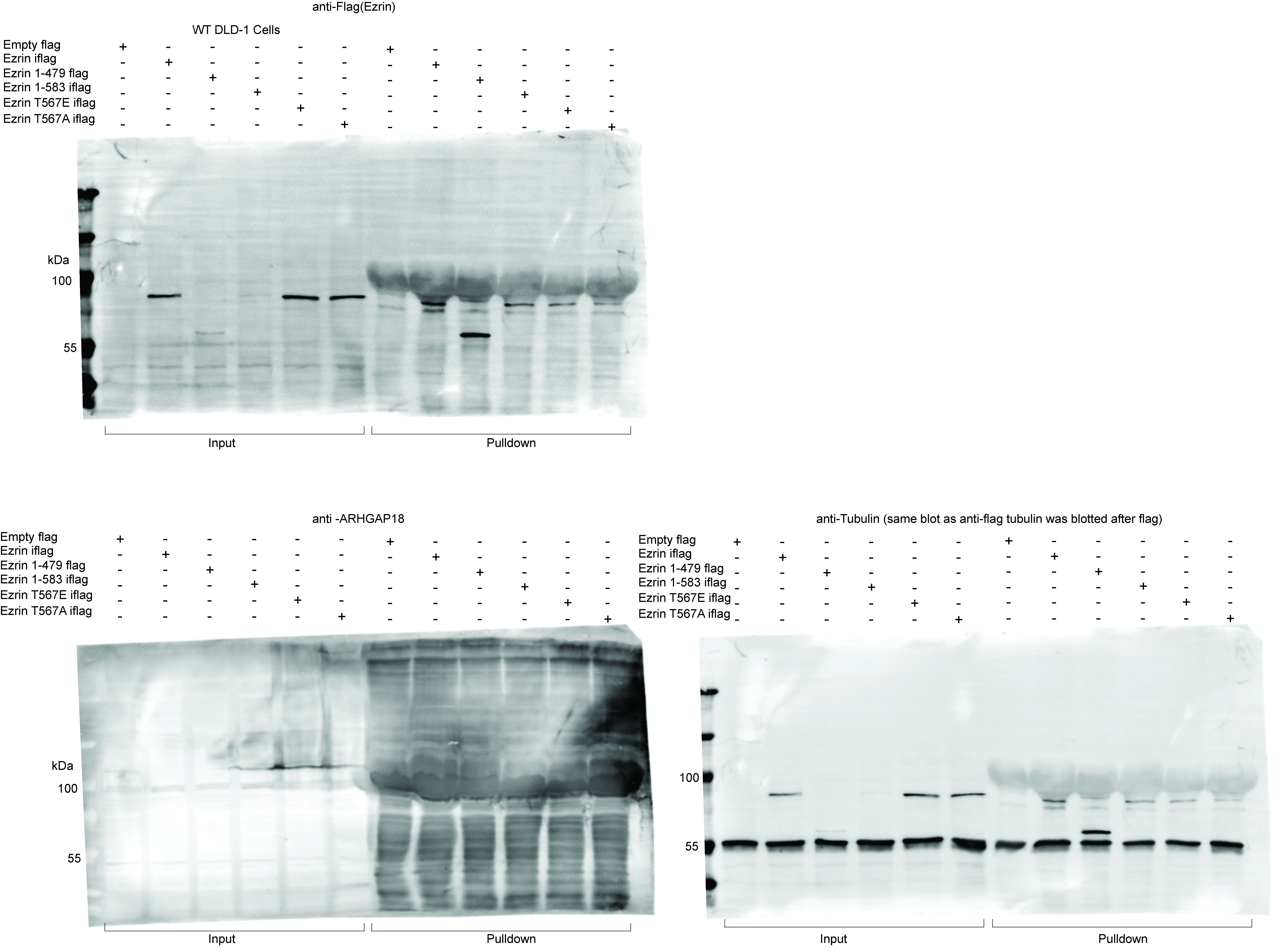

Supplement: Figure 1—figure supplement 2—source data 1. [file elife-83526-fig1-figsupp2-data1.zip › Supplemental Figure S2- source data-1/Supplemental Figure 2 DLD1 Pulldown Source Data.tif]

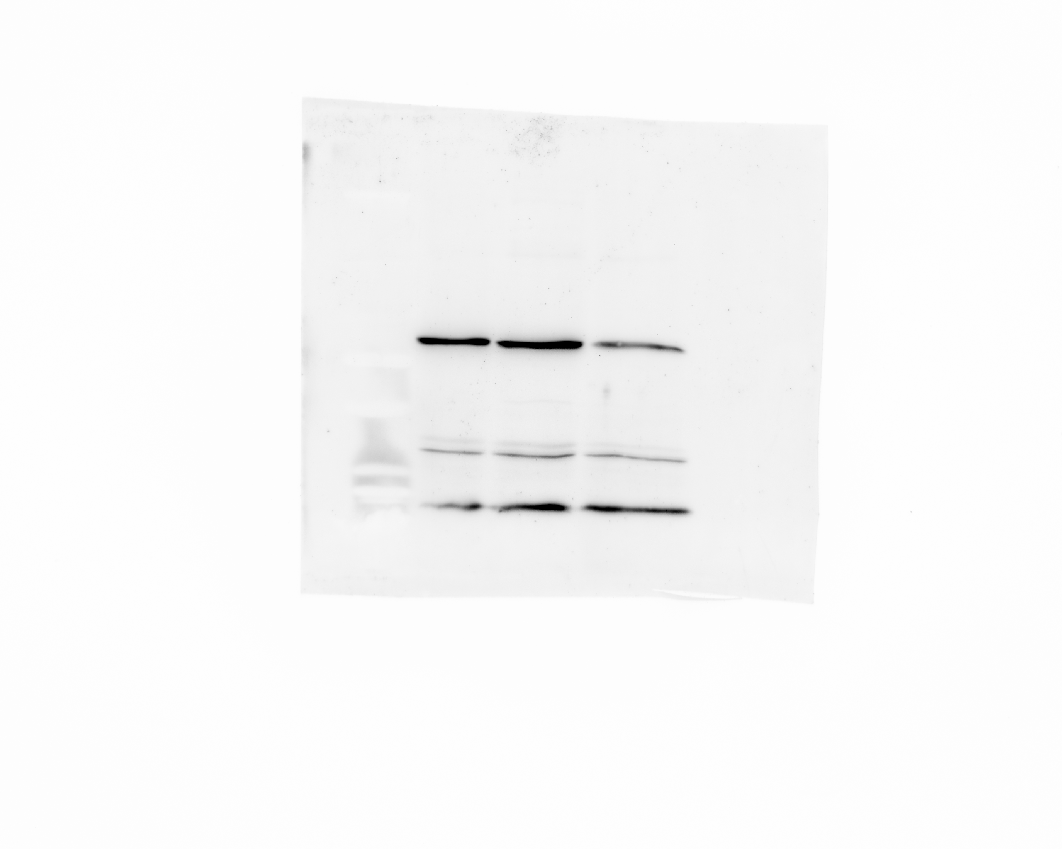

Supplement: Figure 2—source data 1. [file elife-83526-fig2-data1.zip › Figure 2- source data 1/2021-07-23 arhgap ko and over exp t567 IRDye 800CW(IRDye 800CW).tif]

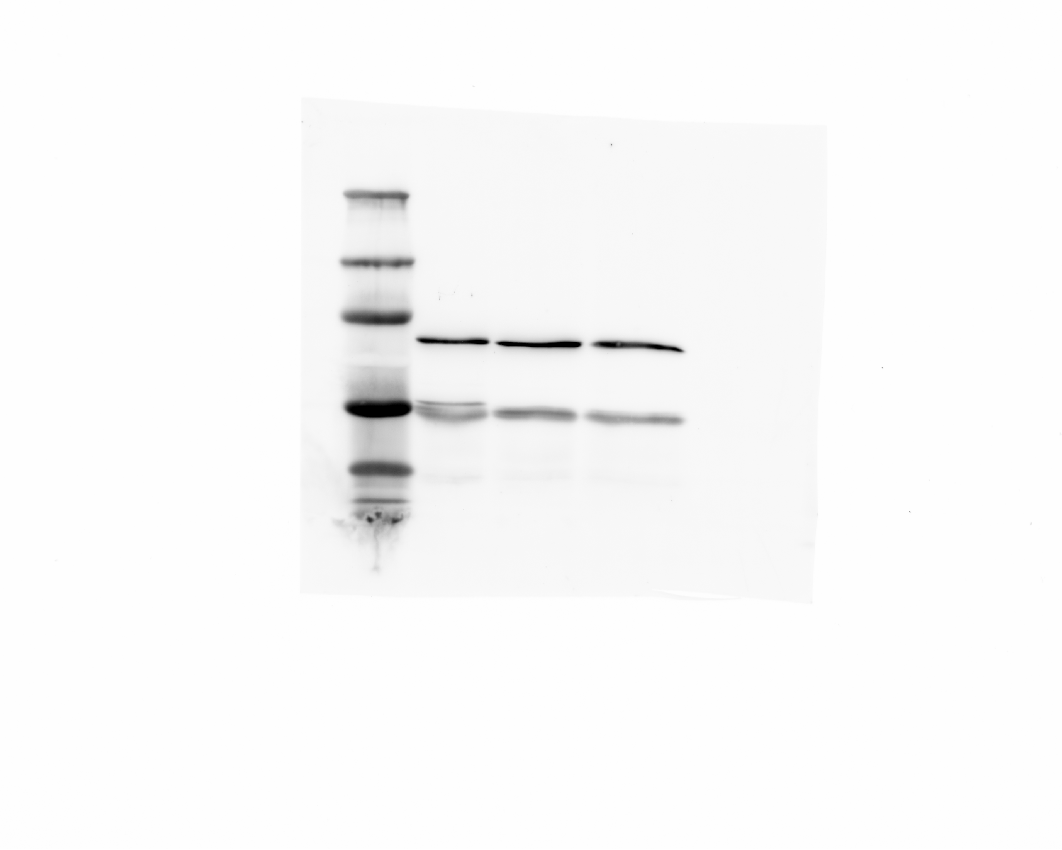

Supplement: Figure 2—source data 1. [file elife-83526-fig2-data1.zip › Figure 2- source data 1/2021-07-2arhgap 18 ko and over exp ezr3IRDye 680RD(IRDye 680RD).tif]

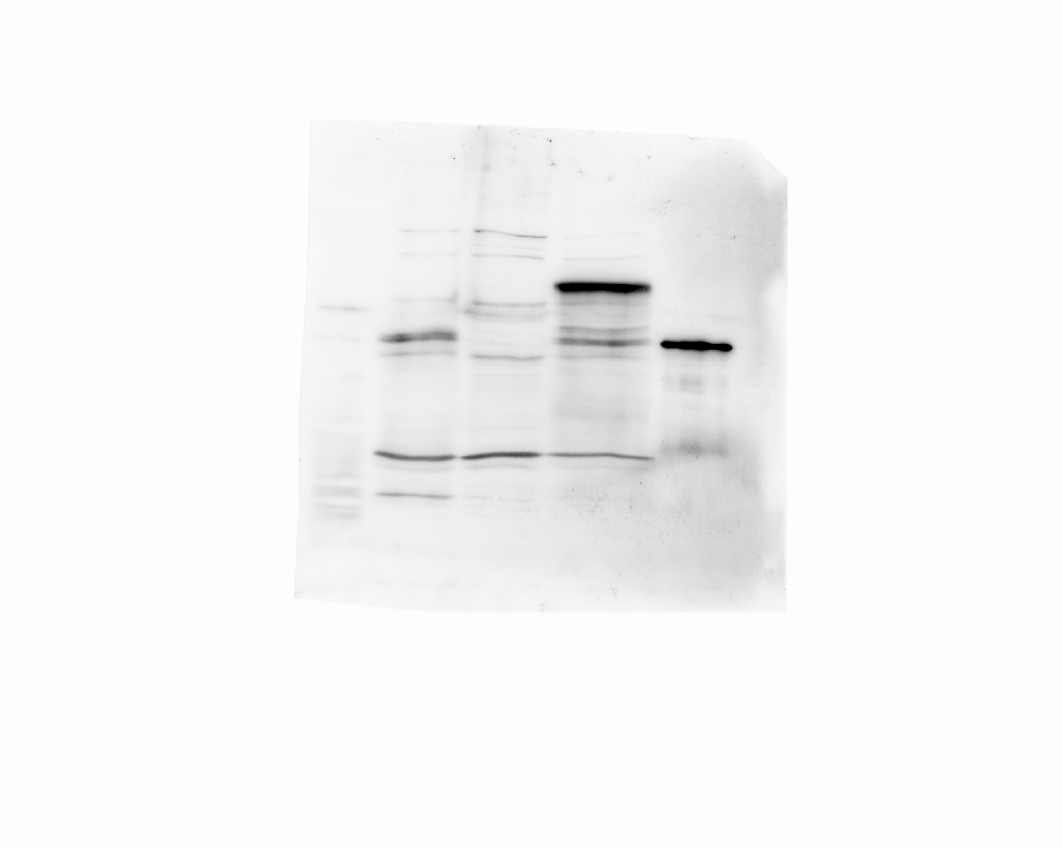

Supplement: Figure 2—source data 1. [file elife-83526-fig2-data1.zip › Figure 2- source data 1/arhgap 18 ko b116_1(IRDye 800CW).tif]

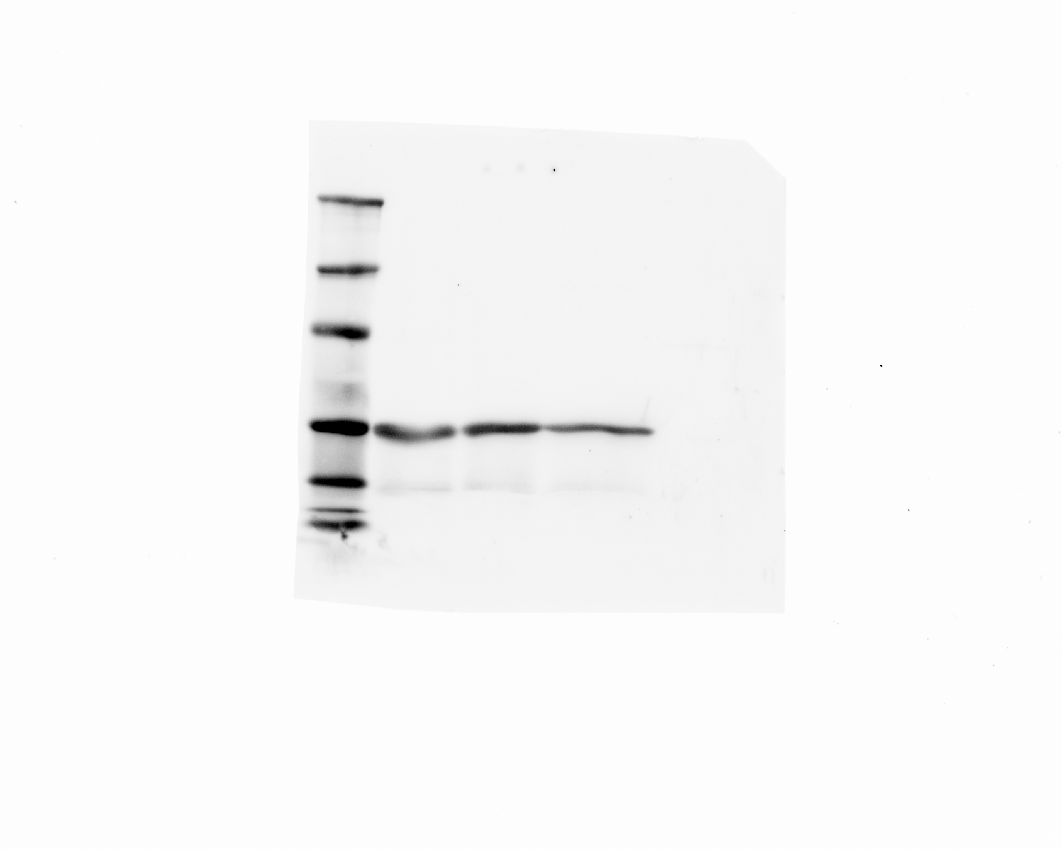

Supplement: Figure 2—source data 1. [file elife-83526-fig2-data1.zip › Figure 2- source data 1/arhgap 18 ko tubulin_2(IRDye 680RD).tif]

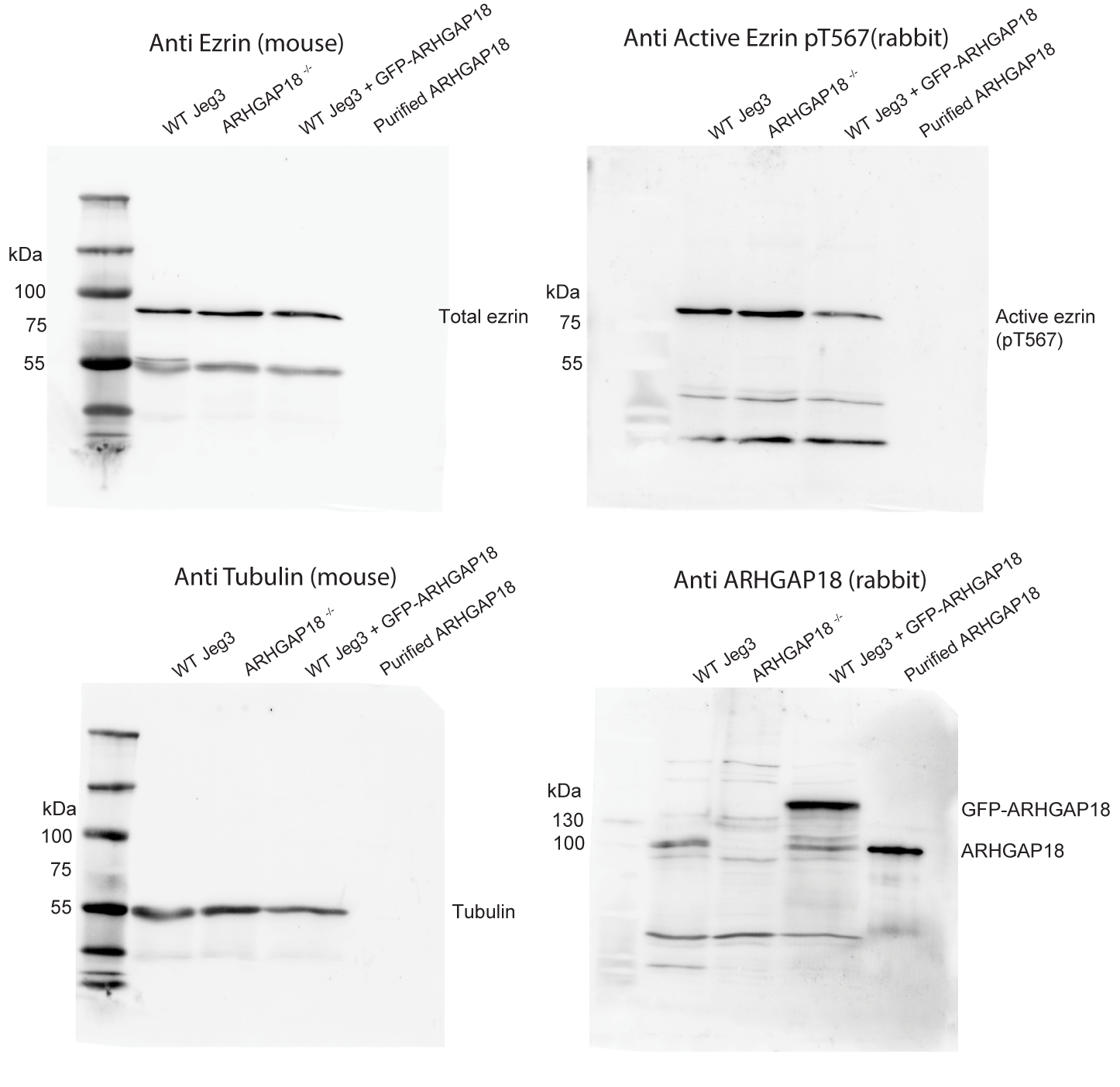

Supplement: Figure 2—source data 1. [file elife-83526-fig2-data1.zip › Figure 2- source data 1/Figure 2 VF-Source Data-02.tif]

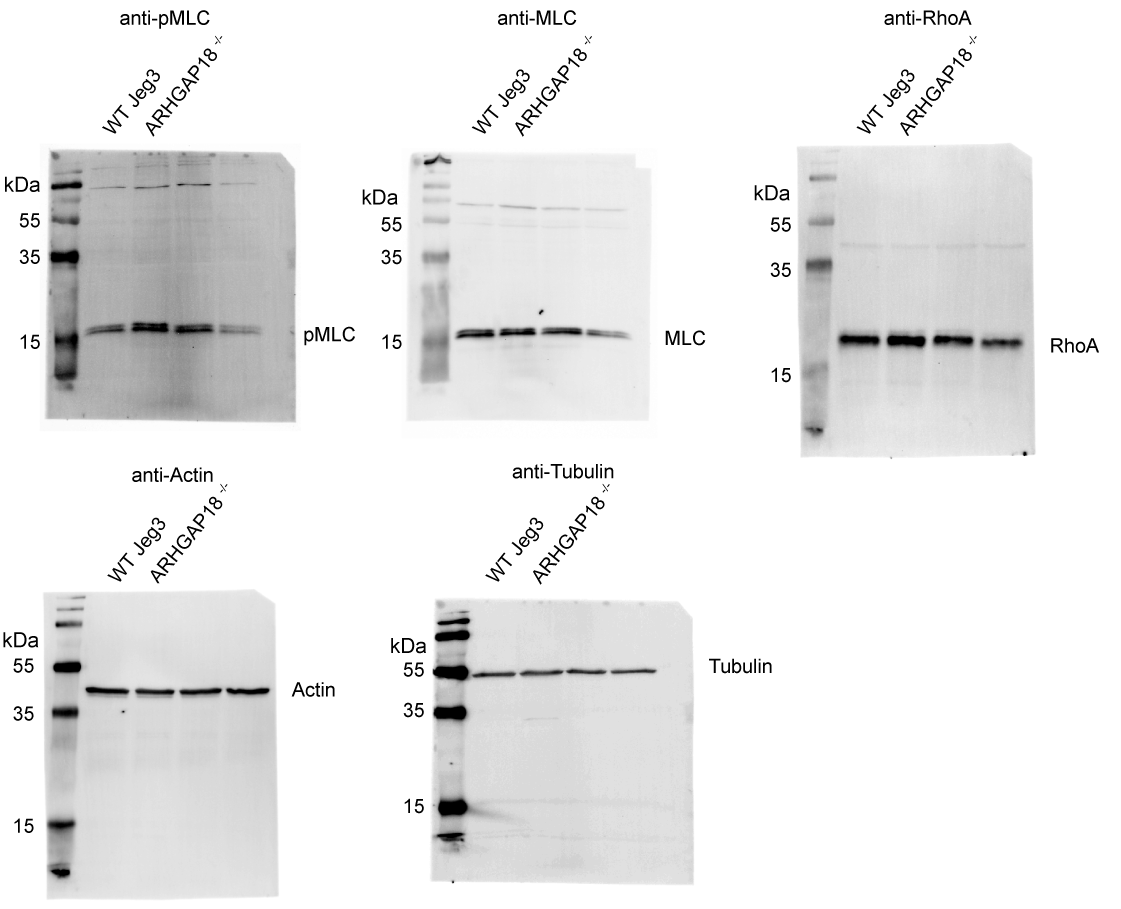

Supplement: Figure 4—source data 1. [file elife-83526-fig4-data1.zip › Figure 4-source data 1/Figure 4 VF Source Data-02.tif]
